# LpA-II:B:C:D:E: a new immunochemically-defined acute phase lipoprotein in humans

**DOI:** 10.1186/s12944-018-0769-6

**Published:** 2018-05-28

**Authors:** John D. Bagdade, Bernd Jilma, Lisa C. Hudgins, Petar Alaupovic, Carrie E. McCurdy

**Affiliations:** 1 Department of Human Physiology, University of Oregon, 122c Esslinger Hall, Eugene, OR 97403 USA; 2 Department of Medicine and Pharmacology, Medical University of Vienna, 1090 Vienna, Austria; 3 Department of Medicine, Weill Cornell Medical College and the Rogosin Institute, New York, NY 10065 USA; 4 Lipid and Lipoprotein Laboratory, Oklahoma Medical Research Foundation, Oklahoma City, OK 73104 USA

**Keywords:** Endotoxin, Inflammation, Lipoprotein subclasses, TGLRP, triglyceride—rich lipoproteins, Innate immunity

## Abstract

**Background:**

Previous studies of lipoproteins in patients with sepsis have been performed on density fractions isolated by conventional ultracentrifugation that are heterogeneous and provide no information about the cargo of apoproteins present in the immunochemically distinct subclasses that populate the density classes. Since apoproteins are now known to have important roles in host defense, we have separated these subclasses according to their apoprotein content and characterized their changes during experimental endotoxemia in human volunteers.

**Methods:**

We have studied apoB- and apoA containing lipoprotein subclasses in twelve healthy male volunteers before and for 8 h after a single dose of endotoxin (ET; 2 μg/kg) to stimulate inflammation.

**Results:**

After endotoxin, TG, TC, apoB and the apoB-containing lipoprotein cholesterol-rich subclass LpB and two of the three triglyceride-rich subclasses (TGRLP: Lp:B:C, LpB:C:E+ LpB:E) all declined. In contrast, the third TGRLP, LpA-II:B:C:D:E (“complex particle”), after reaching a nadir at 4 h rose 49% above baseline, *p* = .006 at 8 h and became the dominant particle in the TGRLP pool. This increment exceeds the threshold of > 25% change required for designation as an acute phase protein. Simultaneous decreases in LpA-I:A-II and LpB:C:E + LpB:E suggest that these subclasses undergo post-translational modification and contribute to the formation of new LpA-II:B:C:D:E particles.

**Conclusions:**

We have identified a new acute phase lipoprotein whose apoprotein constituents have metabolic and immunoregulatory properties applicable to host defense that make it well constituted to engage in the APR.

## Background

The acute phase reaction (APR) is an integral component of host defense that contributes to the initiation, activation, and propagation of events that are integral features of innate immunity [1]. This highly conserved transcriptional response is driven by inflammatory cytokines released from mononuclear cells that activate expression of multiple genes [2] that alter the hepatic secretion of a number of plasma proteins and lipoproteins that have pathophysiological actions [3]. One of these many changes is a rise in triglyceride-rich lipoproteins (TGRLP) that frequently is observed during sepsis. The lipid contents of lipoproteins involved in this response are believed to protect the host both by sequestering and neutralizing microbial toxins and delivering vital nutrients to cells actively engaged in the immune response and tissue repair [4, 5]. The fact that both the structural and exchangeable apoprotein components of lipoproteins are now known to have a wide range of immunoregulatory functions indicates that the protein moieties also serve to protect the host in the presence of infection and inflammation [6–8].

Each lipoprotein density fraction isolated by conventional ultracentrifugation has been considered to be relatively homogeneous. Application of immune-based lipoprotein separation methods has instead revealed a more complex picture. Density fractions in fact are very heterogeneous and contain several discrete subclasses that differ in their apoprotein and lipid composition, function, density, and metabolism and are not detected when conventional density fraction fractions are measured [9].

Separating lipoproteins into immunochemically-defined subclasses is feasible because their major apoproteins are retained during intravascular lipolysis even though their physicochemical properties [10] and distribution within the conventional the density fractions changes. Lipoproteins containing apoB separated in this way have been grouped into two major subclasses – those that are TG-rich (LpB:C, LpB:C:E, LpAII:B:C:D:E) and those that are cholesterol-rich (LpB, LpB:E). Each of these is dispersed widely in VLDL, IDL, and LDL and has differing atherogenic properties and clinical relevance [11]. The apoC-III-containing subclasses LpB:C and LpAII:B:C:D:E for example have been shown to be associated with progression of coronary atherosclerosis [12] and the lipoprotein density classes (VLDL, LDL) containing these subclasses found to predict cardiovascular events [13, 14]. Two major apoA-containing subclasses (LpA-I, LpA-I:A-II) populate the HDL2 and HDL3 subfractions [9].

When endotoxin (bacterial lipolysaccharide: LPS) is released from the cell walls of gram-negative bacteria into the circulation, it binds to the Toll-like receptor (TLR4) on immune cells, which then release cytokines and other inflammatory mediators that activate the APR and initiate the host innate immune response [15]*.* For this reason, endotoxin has become a convenient experimental tool to investigate the APR [16].

Most previous studies of lipoproteins from patients with sepsis [17, 18] and during experimental endotoxemia in human volunteers [19] have been performed on lipoprotein density fractions. Since no information is available about the transport of apoprotein-defined lipoprotein subclasses during the APR, we have characterized these subclasses in a group of normal volunteers following endotoxin exposure.

## Methods

### Study population

Subjects were recruited for study in Vienna, Copenhagen, and New York. The study was performed according to the Declaration of Helsinki. Subjects were informed about the possible risks and discomfort before giving their written consent to participate. The protocol was approved by the Ethical Committee(s) of the Medical University of Vienna, Austria and of Copenhagen and Fredriksberg Communities, DK and by the Institutional Review Boards of Rockefeller University and the Oklahoma Medical Research Foundation. ***Inclusion criteria***: healthy young, non-obese, non-smoking subjects. ***Exclusion criteria***: recent intake of prescription or non-prescription medications.

#### Protocol

All subjects were admitted to the clinical research unit at 0800 after an overnight fast. After voiding, they were placed at bed rest which was continued throughout the entire study period. The twelve male participants in Vienna (age 23 +/− 1 yr.; BMI 23.4 ± 0.5 kg/m^2^; mean ± SEM) received a single i.v. bolus dose of endotoxin containing 2 ng/kg LPS (National Reference). Blood was obtained from these subjects at 0, 2, 4, 6, 8 h. Control subjects received an i.v. bolus dose of saline followed by saline infusions and were studied at three different sites: four subjects were part of the Vienna cohort and had also received endotoxin; an additional six male subjects were studied at the University of Copenhagen (age 24 ± 1 yr.; BMI 23.5 ± 0.8 kg/m^2^); and six subjects studied at Rockefeller University in New York (three males, three females (age 30+/− 1.9 yr.; BMI 25.0 +/− 1.0 kg/m^2^) that participated in earlier studies, in which lipoproteins had been isolated immunochemically and measured during saline infusion [19, 20]. In the subjects from Copenhagen, blood was drawn at 0, 2, 3, 6 h. All samples were processed immediately at each site by centrifugation at 2000 g at 4 °C for 15 min and plasma stored at − 80 °C before analysis. Since there was no difference between the 3 h values in the Copenhagen subjects and the 4 h values in the New York and Vienna control subjects, the results were combined into a single 4 h measurement.

### Analytical methods

Total cholesterol (TC), TG and HDL-Cholesterol (HDL-C) were determined in frozen blood samples [21] and LDL-cholesterol (LDL-C) calculated using the Friedewald formula as previously described [22]. Apolipoproteins (apo) A-I, A-II, B, C-III and E were determined by employing the immunoturbidimetric procedure of Riepponon et al. [23] using corresponding monospecific polyclonal antisera. Quantitative determination of LpB, LpB:C, LpB:E + LpB:C:E and LpA-II:B:C:D:E subclasses was performed by sequential immunoprecipitation of whole plasma by polyclonal antisera to apoAII, apoE and apoCIII, respectively, as previously described [24]. To determine the distribution of apoC-III and apoE between the apoB- and apoA- lipoproteins following endotoxin or saline treatment, the binding of each was measured by electroimmunoassay in heparin soluble (HS; apoA) and heparin precipitate (HP; apoB) fractions and changes in their apoE content expressed as apoE-HS (HDL)/HP (VLDL+ LDL) ratios*.* LpA-I, LpA-I:A-II were measured according to the method of Marz et al. [25]. The between assay CVs for immunoprecipitation with anti-serum to apo CIII was 6–7%.

### Statistical analysis

Data were analyzed by 2-way ANOVA for main effect of time vs. ET treatment with posthoc analyses of significant main effects. A one-way ANOVA was used for comparison of the changes in the apoB-subclasses within each treatment group. In order to better visualize a full 8 h pattern of changes in the TGRLP subclasses in the saline-infused controls in whom these parameters were measured from 0 to 6 h only, regression lines were determined by least squares estimation for the plasma lipids and each subclass from 0 to 6 h and from each line values were estimated at 8 h [26].

## Results

All subjects who received endotoxin manifested one or more of its side effects: typical flu-like symptoms, chills, fever, headache, nausea, and myalgia [27, 28].

### Baseline measures and changes from baseline

**Fig. 1:**
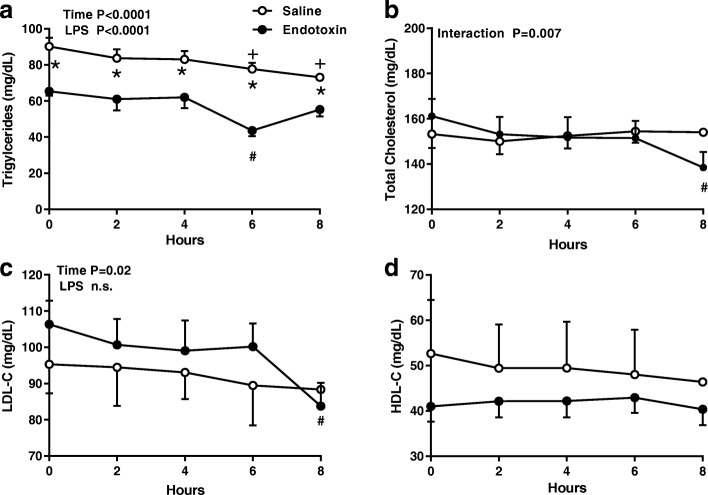
Changes in plasma lipids in response to endotoxin. Fasting plasma (**a**) triglycerides, (**b**) total cholesterol, (**c**) LDL-cholesterol, and (**d**) HDL-cholesterol concentrations (mean +/− SE) were measured in subjects at baseline and for 8 h after an intravenous dose of endotoxin (closed circles, *n* = 7–12) or saline (open circles, *n* = 4–9). Data were analyzed by 2-way repeated measures ANOVA (time x LPS treatment) with Dunnett’s posthoc analysis for time points compared to group baseline with saline (+; *p* < 0.05) or LPS (#; *p* < 0.05). A Sidak’s multiple comparison test was used to compare treatment groups at each time point (*; *p* < 0.05)

**Fig. 2:**
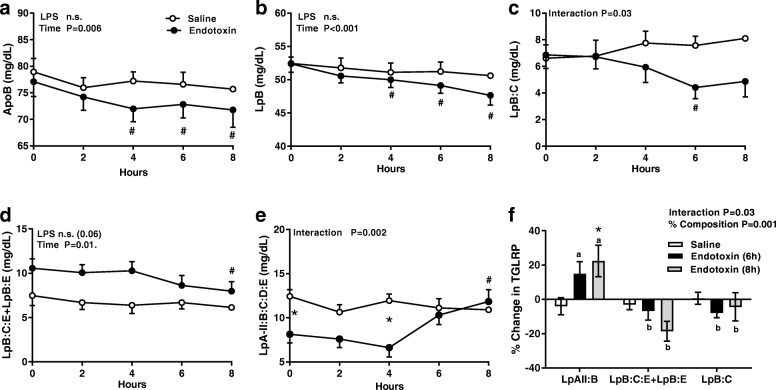
Endotoxin alters the quantity and distribution of apo B-containing lipoprotein subclasses. Plasma (**a**) apoB and apoB-containing lipoprotein subclasses: (**b**) LpB, (**c**) LpB:C, (**d**) LpB:C + LpB:E and (**e**) LpA-II:B:C:D:E concentrations measured at baseline and for 8 h after an intravenous dose of endotoxin (closed circles, *n* = 11) or saline (open circles, *n* = 9). Data were analyzed by 2-way repeated measures ANOVA (time x LPS treatment) with Dunnett’s posthoc analysis for time points compared to baseline in saline (+; *p* < 0.05) or LPS (#; *p* < 0.05) groups. A Sidak’s multiple comparison test was used to compare treatment groups at each time point (*; *p* < 0.05). (**f**) The percent change from baseline in TGRLP subclass distribution at 6 h and 8 h after an intravenous dose of LPS or saline. To compare group changes within each subclass, data were analyzed by 2-way ANOVA (TGLRP composition vs. time) with Tukey post hoc analysis. * *p* < 0.05 compared to saline within subclass. Significant difference (*P* < 0.05) between subclasses letters at 6 or 8 h are marked with different letter

The physical characteristics of the two experimental groups are indicated in the Methods section. At baseline the subjects who received ET had significantly lower TG (*p* = .00*3;* Fig. 1a*)*, LpAII:B:C:D:E (*p* = .016; Fig. 2e), and apoE levels (*p* = .004; Fig. 4a) than the saline controls. The directional changes in TG, TC, LDL-C, and HDL-C, however, were similar in the two groups until 6 h (Fig. 1) when TG in the ET subjects had declined significantly from baseline and was significantly less than the TG in the saline controls (*p* = .0001; Fig. 1a) and HDL-C was lower overall with time in the ET group (*p* = .003).

### ApoB and apoB-subclasses

In the ET group apoB, LpB, and LpB:C declined within 6 h to levels significantly less than baseline (Figs. 2a-c) and their pattern of change differed from those of the saline group. The behavior of LpA-II:B:C:D:E in the ET group, however, differed from that of apoB and other of their apoB- containing subclasses. After declining to a nadir at 4 h, Lp-AII:B:C:D:E then increased progressively over the next 4 h and reached a level at 8 h that was almost two-fold above baseline (*p* = .006)***;*** Fig. 2e). While the plasma TG declined from baseline and the TGRLP pool size contracted in the ET group, the number of Lp-AII:B:C:D:E particles relative to LpB:C and LpB:C:E + LpB:E increased and LpA-II:B:C:D:E: became the predominant TGRLP subclass at 6 h and 8 h (*p* = .001; Fig. 2f). The percentage of each TGRLP subclass in the saline group was unchanged throughout the study.

### ApoA-I and apoA-subclasses

**Fig. 3:**
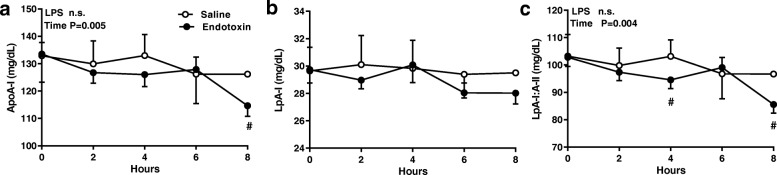
Endotoxin reduces the quantity of apo A-containing particles. The concentration of plasma (**a**) apoA-I and apoA-I containing lipoprotein subclass, (**b**) LpA-I, (**c**) LpA-I:A-II were measured in subjects at baseline and for 8 h after an intravenous dose of endotoxin (closed circles, *n* = 12) or saline (open circles, *n* = 4) groups. Data were analyzed by 2-way repeated measures ANOVA (time x LPS)

From 0 to 6 h, there was no significant change in apoA-I and LpA-I in either group. (Fig. 3a, b). At 8 h, however, both apoA-I and LpA-I:A-II in the ET subjects declined significantly from baseline (*p* = .0001). Since LpA-I levels remained stable from 6 to 8 h, these findings indicate that the decrease in apoA-I was due to a specific decline in the LpA-I:A-II subclass.

### Distribution of apoE and apoC-III

**Fig. 4:**
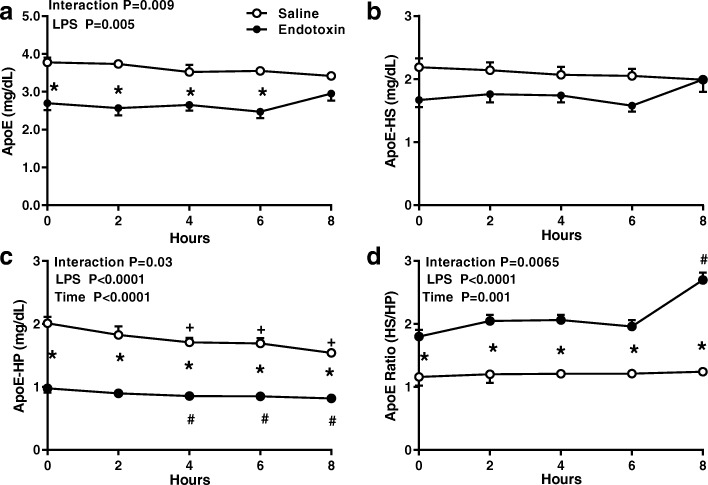
Endotoxin alters distribution of ApoE among plasma lipoproteins. The concentration of (**a**) apoE in plasma, (**b**) apoE associated with HDL (ApoE heparin soluble [HS]), (**c**) apoE associated with apoB-containing lipoproteins (ApoE-heparin precipitate [HP]) was measured at baseline and for 8 h after an intravenous dose of endotoxin (closed circles, *n* = 11) or saline (open circles, *n* = 4). (**d**) Changes in distribution of apoE in heparin soluble (HS) and heparin precipitate (HP) is expressed as the apoE HS/apoE HP ratio. Data were analyzed by 2-way repeated measures ANOVA (time x LPS treatment) with Dunnett’s posthoc analysis for time points compared to baseline in saline (+; *p* < 0.05) or LPS (#; *p* < 0.05). A Sidak’s multiple comparison test used to compare treatment groups at a single time point (*p < 0.05)

Apo E concentration at 0 h in the ET subjects was significantly lower than in the saline-treated controls and levels in both groups were stable until 6 h after ET. At 8 h, however, total apoE in the ET group trended upward from baseline and this small increment (+ 10%) was reflected by increases in the apoE content of HDL (apoE-HS; Fig. 4b) which rose significantly (+ 27%; *p* = .01) above baseline and in the apoE HS/HP ratio (+ 40%; *p* = .0001; Fig. 4d). In both the saline and ET-treated subjects, the apoE content of apoE-HP (VLDL+LDL) declined from baseline from 4 to 8 h (Fig. 4c).

**Fig. 5:**
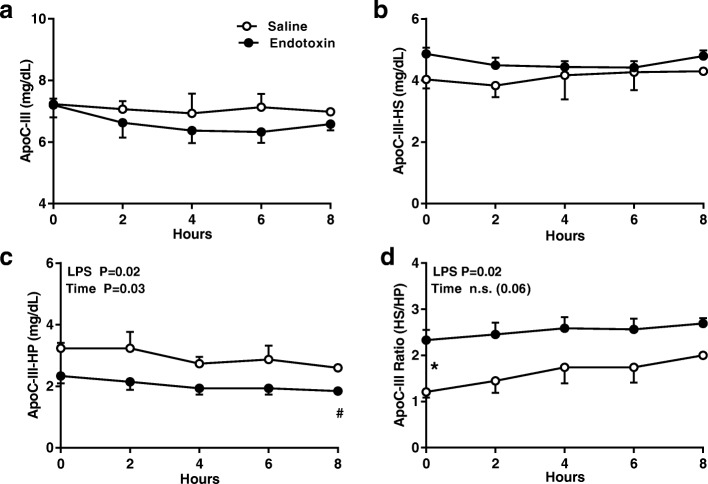
Endotoxin does not significantly change the distribution of ApoC-III among plasma lipoproteins. The concentration of (**a**) apo C-III in plasma, (**b**) apo C-III associated with HDL (C-III heparin soluble [HS]), (**c**) apo C-III associated with apo B-containing lipoproteins (C-III heparin precipitate [HP]) was measured at baseline and for 8 h after an intravenous dose of endotoxin (closed circles, *n* = 11) or saline (open circles, *n* = 4). (**d**) Changes in the distribution of apo C-III in HS and HP expressed as the C-III HS/C-III HP ratio (apo C-III R) after the intravenous injection of endotoxin (LPS). Data were analyzed by 2-way repeated measures ANOVA (time x LPS treatment) with Dunnett’s posthoc analysis for time points compared to baseline in saline (+; *p* < 0.05) or LPS (#; *p* < 0.05). A Sidak’s multiple comparison test was used to compare treatment groups within each time point (*; *p* < 0.05)

ApoC-III levels at baseline were similar in the two groups (Fig. 5a), thereafter declining in plasma and in the apoB-containing lipoproteins (apoC-III HP; Fig. 5c) and increasing at 8 h in HDL (apoC-III HS; Fig. 5b) in a pattern similar to that of apoE. These changes, however, were not statistically significant.

## Discussion

Disturbances in plasma lipids have been observed for many years in patients during sepsis [17, 18, 28]. In the most comprehensive sepsis-related study of lipoprotein transport to date, sequential changes in the concentration of lipoprotein density fractions were measured and correlated with levels of cytokines, inflammatory markers, and acute phase reactants during experimental endotoxemia in human volunteers [19]. Hudgins et al. [19] observed an early and rapid increase in TG and VLDL lipids that peaked at 3 h and was synchronous with maximum levels of IL-6 and TNF-alpha.

Previously, we examined immunochemically-defined lipoprotein subclasses in human volunteers during an IL-6 infusion to investigate lipoprotein subclasses during systemic inflammation [20]. In that project, we found that the concentration of the TGRLP subclasses LpB:E + LpB:C:E,which are distributed in the apoB-containing VLDL, IDL, and LDL density classes increased significantly at 30 min and 60 min with no change in plasma TG. Since IL-6 is only one of several inflammatory mediators released during the acute phase reaction [29], this observation suggested that simulating inflammation with endotoxin may impact the transport of this and other immunochemically-separated lipoprotein subclasses. Our current results confirm this hypothesis.

Except for the lack of an early increase in plasma TG, the directional changes we observe in the major plasma lipids and apoB in the ET group from 0 to 6 h resemble those described by Hudgins et al. [19]. As previously reported, we too find that individual TG responses during systemic inflammation and sepsis are variable [1, 18, 30]. While the changes in the apoB-subclasses from 0 to 4 h did not differ in our two experimental groups, their subsequent responses differed significantly. Notably, as the plasma TG and the TGRLP subclasses LpB:C and LpB:C:E continued to decline in the ET subjects, their LpA-II:B:C:D:E (LpA-II:B complex) particle number increased progressively and this particle which normally is only a minor component (7%) of the TGRLP pool [9, 10] became the most abundant TGRLP particle.

By increasing more than 25% above its baseline value (+ 27% at 6 h and + 48% at 8 h), the LpAII:B complex particle meets the definition of an acute phase reactant [1, 29] and therefore is a previously unrecognized positive acute phase protein. Even though the overall changes in plasma TG and TGRLP pool size after endotoxin were modest, we believe that the increase in number of this specific particle is biologically significant because it contains several multifunctional apolipoproteins that have immunomodulatory properties. Therefore, the fact that these particles increase in number during inflammation makes it likely that they contribute actively to host defense. Alaupovic first identified the LpAII:B complex particle in the plasma of patients with Tangier disease and showed that it differed metabolically from other TGRLP by being lipolysis resistant and a poor substrate for LPL [31]. More recent kinetic studies showing that it has a prolonged residence time in plasma are consistent with his earlier observations [32].

The concentration of most acute phase proteins is regulated by APR genes [33] at the transcriptional level through changes in hepatic production [34]. The alterations we observe in lipoproteins, however, are too rapid to be ascribed to changes in production. Rather, our findings suggest that changes in LpA-II:B particle number was a post-translational event involving the coordinated activity of lipases and lipid transfer proteins that normally play integral roles in the remodeling of TGRLP and HDL [32]. Indeed, Alaupovic et al. speculated earlier that LpA-II:B particles were formed in plasma by the transfer of apoA-II from the HDL subclass LpA-I:A-II particles to LpB:C:E [31]. The concomitant increase we observe in LpA-II:B and decline in both the LpA-I:A-II and LpB:C:E + LpB:E from 4 to 8 h after endotoxin supports this mechanism.

Based on its apoprotein content and kinetic behavior [32], we suggest that the LpAII:B complex particle is well suited to engage in the APR and plays an important role in host defense. Being resistant to lipolysis and having a prolonged residence time in plasma during inflammation may be useful because this property enhances its capacity to deliver nutrients and apoproteins to immune cells that support their activation [35]. For example, apoA-II can upregulate and then modulate the host response during sepsis [36]. Although better known for its role in cholesterol transport and macrophage biology, apoB-100 also can act as an immune suppressor by limiting the release of cytokines [37]. Because LpA-II:B:C:D:E, has apoB-100 as its major structural apoprotein, it would under normal circumstances facilitate its internalization by LDL B,E receptors in both hepatic and extrahepatic tissues throughout the body. During infection, however, LDL receptors are down-regulated in the liver and upregulated in macrophages [38], changes thought to benefit the host by promoting the uptake of apoB-containing subclasses by immune cells. Not surprisingly, two of the three apoC isoforms present on LpA-II:B:C:D:E also are involved in host defense (7). Quite apart from their regulatory roles in lipoprotein transport [39], apoC-I has been shown to enhance the inflammatory response to LPS [40] and apoC-III to actively participate in the inflammatory components of atherosclerosis development [41].

Despite apoD being structurally dissimilar from other apolipoproteins [42], it too has immunoregulatory, anti-stress, and antioxidant properties that contribute to host defense [43]. Alaupovic suggested earlier that apoD was acquired from LpA-I:A-II HDL particles when LpAII:B complex particles are formed from the interaction of LpA-I:A-II with LpB:C:E [31]. While apoE is a key ligand that facilitates transport of the apoE-containing apoB subclasses, most apoE (50–75%) in humans is associated with circulating HDL [44]. Like many other HDL constituents [6], apoE is involved in both immunoregulation and host defense [45]. During infection, for example, apoE can multi-task and simultaneously neutralize LPS and modulate lipoprotein trafficking [46].

Since atherosclerosis is accelerated in a number of chronic inflammatory diseases [35], it is relevant to the present study that LpA-II:B:C:D:E particle number is increased and associated with progression of atherosclerosis in patients with rheumatoid arthritis [47]. Because our study indicates that this particle is an acute phase reactant closely linked to inflammation, it seems likely that it poses a similar risk in patients with Tangier disease who also develop cardiovascular disease prematurely [48].

The behavior we observe of the two major immunochemically-defined HDL subpopulations, LpA-I and LpA-I:A-II, after endotoxin add to the growing body of information about the changes that HDL undergoes during inflammation [49, 50]. Despite the extensive remodeling of HDL surface and core constituents and the decline in HDL-C and apoA-I that is known to occur during the APR [19, 50], we show that the same percentage distribution of 25% LpA-I and 75% LpA-I:A-II present at baseline was maintained for 8 h after endotoxin.

We also provide preliminary information about the transport of the exchangeable apoproteins apoE and apoC-III during the APR. For the first 6 h, apoE associated with HDL and the apoB lipoproteins (VLDL, IDL, and LDL) declined to a similar degree in both the ET and saline groups. By 8 h, however, the apoE present in HDL in the ET group increased 28% above baseline as first reported in septic patients and identified as an acute phase protein by Li et al. [51]. In contrast to most other acute phase proteins that involve de novo hepatic synthesis, these workers found that the increase in apoE during sepsis resulted from a combination of inhibition of apoE degradation and down-regulation of hepatic LDL receptors [38, 51].

The movement of apoC-III from the apoB lipoproteins (HP) to HDL (HS) resembled that of apoE but the magnitude was small, the number of observations limited, and the changes were not statistically significant. In light of heightened awareness of the proinflammatory properties of apoC-III and the key role that it and other HDL-associated proteins having immunomodulatory properties (A-IV, C-III, C-IV, L-I, M, F, H, J [clusterin]) play in host defense, their distribution among the HDL subclasses and fate during the APR require further study [6].

The strength of our study is that we have employed an underutilized immunochemical method of measuring lipoproteins to demonstrate for the first time changes in the TGRLP subclasses during inflammation that are not revealed by conventional methods of lipoprotein isolation.

A limitation of this study is that our measurements are limited to the first 8 h after ET. While a longer period of observation would be desirable, it was still possible within this time to discern changes in lipoproteins during the APR with a new level of precision and to identify LpA-II:B:C:D:E as a new acute phase reactant. Longer studies are needed to determine the duration of LpAII:B:C:D:E elevation, the extent to which it and other immunochemically-defined lipoprotein subclasses contribute to the APR, and the degree to which changes in their concentration correlate with inflammatory mediators.

Other concerns are that our control subjects were studied at different sites, their 8 h data was incomplete, and some of their baseline lipid measures differed from those of the ET group. While demographic differences likely account for the disparity in baseline lipids, the changes exhibited in their plasma lipids during saline infusion correspond closely to those reported by Hudgins under identical experimental conditions [19]. Importantly, neither these site differences or our estimating 8 h TG and TGRLP subclass values influenced our conclusions.

## Conclusion

Employing an underutilized immunochemical method of measuring lipoproteins according to their apoprotein content, we have identified a new acute phase lipoprotein whose apoprotein constituents have metabolic and immunoregulatory properties applicable to host defense that make it well constituted to engage in the APR.

## Abbreviations

APR: Acute phase response
BMI: Body mass index
ET: Endotoxin
HDL: High density lipoprotein
IDL: Intermediate density lipoprotein
LDL: Low density lioprotein
LPL: Lipoprotein lipase
LPS: Bacterial lipopolysaccharride
TG: Triglyceride
TGRLP: Triglyceride-rich lipoprotein
TLR: Toll-like receptor
TNF: Tumor necrosis factor
VLDL: Very-low density lipoprotein
